# Examining the effects of the histone methyltransferase inhibitor BIX-01294 on histone modifications and gene expression in both a clinical population and mouse models

**DOI:** 10.1371/journal.pone.0216463

**Published:** 2019-06-11

**Authors:** Kayla A. Chase, Benjamin Feiner, Marcia J. Ramaker, Edward Hu, Cherise Rosen, Rajiv P. Sharma

**Affiliations:** 1 Department of Psychiatry, University of Illinois at Chicago, Chicago, IL, United States of America; 2 Jesse Brown Veterans Affairs Medical Center, Chicago, IL, United States of America; 3 Department of Psychiatry, University of California, La Jolla, CA, United States of America; Chiba Daigaku, JAPAN

## Abstract

Schizophrenia has been consistently characterized by abnormal patterns of gene down-regulation, increased restrictive chromatin assemblies, and reduced transcriptional activity. Histone methyltransferase (HMT) mRNA and H3K9me2 levels are elevated in postmortem brain and peripheral blood cells of persons with schizophrenia. Moreover, this epigenomic state likely contributes to the disease, as HMT levels correlate with clinical symptomatology. This manuscript sought to establish the potential therapeutic value of the HMT inhibitor BIX-01294 (BIX). Human peripheral mononuclear cells (PBMC) from 24 individuals with schizophrenia and 24 healthy individuals were cultured in the presence of BIX (5uM or 10uM). Mice were given once daily intraperitoneal injections of BIX (0.5 or 1mg/kg) for one week. Cultured cells, mouse cortex, or striatum was harvested, RNA extracted and RT-PCR conducted for several schizophrenia candidate genes: IL-6, Gad1, Nanog, KLF4, Reln, and Bdnf9a. Total H3K9me2 levels were measured using western blot while H3K9me2 binding to selected genes of interest was conducted using chromatin immunoprecipitation (ChIP). Neuronal subtype-specific BDNF conditional knockdown was conducted using the cre/lox system of mutant animals. Treatment with BIX decreased H3K9me2 and increased selected mRNA levels in cultured PBMCs from both normal controls and participants with schizophrenia. In mice, peripheral administration of BIX decreased cortical H3K9me2 levels and increased schizophrenia candidate gene expression. In BDNF conditional knockdown animals, BIX administration was able to significantly rescue Bdnf9a mRNA levels in ChAT and D1 Bdnf conditional knockdown mice. The results presented in this manuscript demonstrate a potential for further research into the clinical effectiveness of histone modifying pharmacology in the treatment of schizophrenia.

## Introduction

Schizophrenia can be a chronic and debilitating mental disorder that impacts psychological, social, and cognitive processes across the lifespan [1]. It is characterized by positive symptoms, including delusions and hallucinations, negative symptoms, including social withdrawal and anhedonia, and cognitive symptoms, including disordered thoughts and poor memory and concentration [2]. Cognitive symptoms are present early in the disease, are largely resistant to current pharmacology, and are an important predictor of clinical outcomes. In the central nervous system, Brain Derived Neurotropic Factor (BDNF) is a neurotrophin that is heavily involved in synapse regulation, learning and memory. BDNF is also a candidate gene, with decreased levels found in both peripheral serum and brain from participants with schizophrenia. Deficits in BDNF functioning could lead to the cognitive dysfunction present in schizophrenia [3]. Additionally, BDNF levels are modified through epigenetic mechanisms, including histone acetylation [4,5], methylation [6], and DNA methylation [7,8].

Epigenetics is the study of environmentally induced changes in gene expression that arise from post-transcriptional modifications to chromatin, which consists of both DNA and its packaging proteins called histones [9]. It is through this mechanism that environmental events, such as experience, learning, and trauma can alter patterns of gene expression that can persist for the lifetime of an organism. Epigenetic modifications result in protein assemblies that are conceptualized as transcriptionally ‘permissive’ or ‘restrictive.’ Permissive assemblies have a relaxed chromatin structure, with increased accessibility of regulatory proteins to the DNA, whereas restrictive assemblies effectively seal the gene from access and decrease transcription [10].

Schizophrenia has been consistently characterized by abnormal patterns of gene down-regulation, increased restrictive chromatin assemblies and reduced transcriptional activity [11]. Participants with schizophrenia exhibit lower peripheral levels of acetylated histones (a permissive modification) and higher levels of the enzymes that remove these acetyl groups (histone deacetylases) compared to control participants [12–14]. However, clinical trials utilizing the histone deacetylase inhibitor valproic acid (VPA) found that histones from participants with schizophrenia were less responsive to VPA treatment compared to participants with bipolar disorder [15]. It is our hypothesis that this lack of response in participants with schizophrenia following VPA treatment may be due to increased histone deacetylases or even increased histone methylation at that specific residue.

Di-methylation of H3K9 (H3K9me2) is a repressive histone mark associated with decreased promoter activity. This modification is predominantly catalyzed by two histone methyltransferases (HMT), G9a and GLP, which function as a heteromeric complex [16–18]. HMT mRNA and H3K9me2 levels are elevated in lymphocytes and postmortem brain tissue in participants with schizophrenia. Moreover, this epigenomic state likely contributes to the disease, as HMT levels correlate with clinical symptomatology [19–21]. These durable and repressive methyl modifications may explain why manipulating permissive modifications, such as acetylation, do little to change the epigenetic landscape in participants with schizophrenia [22,23].

Histone methylation and the enzymes that add these modifications are critically involved in cognitive processes, including intellectual disability, addiction and cognitive decline [24]. G9a and GLP inhibition in the entorhinal cortex enhances contextual fear conditioning [25]. G9a conditional knockdown increases cocaine place preference acquisition, while G9a overexpression decreases it. Overrexpression of G9a is also able to significantly decrease cocaine-induced increases in Bdnf and Fos expression in the cortex [26]. In Huntington’s patients, H3K9me2 levels are increased in both human and mouse models, with pharmacologically induced decreases demonstrating neuroprotective effects [27].

Given that schizophrenia is associated with increased levels of both H3K9me2 and HMT mRNA [20], and these repressive insults are associated with both schizophrenia symptoms, and cognitive and learning deficits, this manuscript sought to establish the potential therapeutic value of the G9a and GLP inhibitor BIX-01294 (BIX) as a means of relieving restrictive epigenome characteristic of schizophrenia. This study consisted of three aspects. Firstly, we report that BIX decreases H3K9me2 and increases mRNA levels in cultured human peripheral mononuclear cells (PBMC) from both normal controls and participants with schizophrenia. Secondly, conducted a feasibility study to examine the effect of peripherally administered BIX to determine the optimal dose for animal survival and alterations of H3K9me2 levels in the brain. In this cohort of animals, we demonstrated that peripheral administration of BIX decreases H3K9me2 levels and increases candidate gene expression in mouse cortical brain extracts. Finally, we examined specific neuronal cell types in the mouse striatum, a region critically involved in schizophrenia, specifically the cognitive and negative symptoms [28,29]. We utilized a heterozygous, conditional knockout of Bdnf on allele using the Cre/loxP system, as BDNF is reduced in participants with schizophrenia, and increasing protein levels may have beneficial clinical outcomes [30]. BIX administration significantly rescued Bdnf9a mRNA expression in ChAT and D1 Bdnf conditional knockdown mice. Taken together, these results demonstrate a potential for further research into the clinical effectiveness of histone modifying pharmacology in the treatment of schizophrenia.

## Materials and methods

### Human participants

The sample included 24 individuals with schizophrenia and 24 healthy individuals who were recruited from the University of Illinois at Chicago (UIC) medical center and surrounding community. All human studies were approved by the University of Illinois at Chicago Office for the Protection of Research Subjects University of Illinois Internal Review Board #1 Biomedical and Biological Science Research, under protocol #2012–0113. All clinical investigations were conducted according to the principles expressed in the Declaration of Helsinki. Written informed consent was obtained before beginning any research procedures. In order to account for barriers to understanding research procedures, all participants, regardless of illness status, education level, etc., were subjected to a longer, discussion based informed consent process, in which there was a heavy emphasis on open-ended questions with additional opportunities to review and discuss the procedures conducted [31]. If the participant was unable to respond to open-ended questions about research procedures conducted, participation was terminated. Experienced diagnosticians (MD or PhD) assessed participants using the Structured Clinical Interview for DSM Disorders (SCID) interview, DSM-IV-TR criteria and all available collateral information. Diagnosticians established a consensus before final assignment to diagnostic group. All participants were between 21–65 years of age and in good physical health, with no reported active infections. Exclusion criteria for all participants included: a history of neurological disease or head trauma, lifetime history of substance/alcohol dependence or recent substance abuse, and pregnancy for women. Exclusionary criteria for control participants also included a major Axis I disorder or a known first-degree familial history of psychosis. Participant demographics are listed on Table 1.

**Table 1:** Demographics comparing healthy controls and schizophrenia cases in PBMC samples.

Demographic	Healthy Controls	Participants with Schizophrenia
Sex (M/F)	17/7	17/7
	t = 0.00; *p* = 1.0	
Age (Mean ± SD)	35.9±10.8	35.6±13.8
	t = 0.88; *p* = 0.93	
Race (%)		
Caucasian, non-Hispanic	6 (25)	1 (4.5)
Black, non-Hispanic	11 (46)	16 (73)
Asian or other Pacific Islander	4 (17)	1 (4.5)
Hispanic	3 (12)	4 (18)
	F(1,47) = 1.11; *p* = 0.51	

Means and standard deviations of participant demographics comparing healthy controls and participants with schizophrenia in PBMC samples. Statistical differences between the various parameters were determined by a Student’s t-test, and indicated where performed.

At the time of sampling, 67% (n = 16) of the participants with schizophrenia were evaluated while hospitalized on the inpatient psychiatric unit and 33% (n = 8) were evaluated in the psychiatric outpatient clinic. Prescribed antipsychotic medication for all participants is as follows: Haloperidol = 1, Fluphenazine = 1, Risperidone = 9, Olanzapine = 2, Quetiapine = 3, Aripiprazole = 2, Lurasidone = 2, and Paliperidone = 1 (a total of n = 21). Three participants were unmedicated at the time of the blood draw. While there was no BIX administration to subjects, and the drug was added to *in vitro* culture, we wanted to control for any alterations due to psychotropic medication currently administered to the participant. We did not find any differences in any biochemical measurements studied when controlling for antipsychotic medication regimen.

### Peripheral blood mononuclear cell (PBMC) extraction and culture

A blood sample was obtained by sterile venipuncture and collected in 0.5M Ethylene-diamine-tetraacetic acid (EDTA), pH 8.0. Blood sample was diluted 1:1 with Hanks Balanced Salt Solution (HBSS without calcium; GIBCO 14170–112), and carefully layered over Ficoll-Paque (GE Healthcare Life Sciences) in a 1:1 ratio, and centrifuged at 1,800RPM for twenty minutes at 10°C. The resulting cream-colored opaque middle interface was collected, diluted with HBSS, and pelleted at 2,000RPM for ten minutes at 10°C.

PBMCs were cultured at a concentration of 1X10^6^ cells/mL in complete media consisting of: RPMI 1640, penicillin/streptomycin, l-glutamine, sodium pyruvate, nonessential amino acids, and fetal bovine serum, and incubated in 5% CO^2^ at 37°C for 24 hours in vehicle or 5uM or 10uM of BIX-01294 (BIX). Cell viability was measured both at treatment and harvest via cell counts obtained from a representative well, per condition, following 3 minute incubation in a media/trypan blue mixture, staining for nonviable cells [32–35]. After culture, PBMCs were collected, pelleted at 2,000RPM for ten minutes at 10°C and frozen in -80°C until further processing [20,36,37].

### Animal housing

All procedures were approved by the The Animal Care Committee (ACC) through the Office of Animal Care and Institutional Biosafety (OACIB) at the University of Illinois at Chicago. Approval #: 18–091. The first cohort of animals were treated as a “feasibility study” to determine the optimal dose of peripherally administered BIX on animal survival and H3K9me2 deceases in brain tissue. For this feasibility study, adult male Swiss Albino mice (Harlan; 20-25g) were used (n = 5 per condition), and were given a once daily intraperitoneal injection (i.p.) of BIX-01294 (0.5 or 1 mg/kg), or vehicle (saline) for one week [38]. BIX was dissolved in a volume of saline solution corresponding to 0.1mL/10g body weight. For the BDNF study, three different strains of mice were used utilizing a cre/lox breeding pattern for gene conditional knockdown. Homozygous Bdnf floxed mice (Bdnftm3Jae/J; Jax#004339) were bred to one of two heterozygous *cre* mouse lines: ChAT-*cre* (B6;129S6-Chattm2(cre)Lowl/J; Jax#006410) and D1R-*cre* (b6;129-Tg(Drd1a-cre)120Mxu/Mmjax; MMRRC#037156-JAX) (n = 15 per condition per genotype). This resulted in heterozygous Bdnf conditional knockdown animals, with one copy of the Bdnf gene intact. Thus all animals still expressed some level of Bdnf. Adult mice were given a once daily intraperitoneal injection (i.p.) of BIX-01294 (1 mg/kg), selected based on the initial feasibility study, or vehicle (saline) for one week. For both studies, all animals were housed under a light-dark cycle of 14:10 hours, and food and water was available *ad libitum*. Weights were recorded daily (feasibility study: S1 Fig). Twenty-four hours after the last injection, animals were euthanized with CO_2_, followed directly by cervical dislocation. For the feasibility study the cortex was collected. From the ChAT/BDNF and D1/BDNF mice whole striatum was collected. Brain sections were quickly dissected and snap frozen in 2-Methylbutane (Fisher #03551–4) and stored at -80°C until used for biochemical analysis. Each day during treatment animals were observed for signs of distress, including reductions in grooming or weight.

### Protein extraction and enzyme-linked immunosorbent assay (ELISA)

Total protein was extracted from human PBMCs and mouse brain using the Guanidinium thiocyanate/-phenol-chloroform extraction method (Life Technologies), and levels were quantified by Bradford assay. H3K9me2 (Active Motif #53109) and total H3 protein (Active Motif #53110; for normalization purposes) were measured utilizing their respective ELISA kits, per manufacturer instruction.

Each sample was run in duplicate for each analyte. We used a 96-well format, and each plate was loaded with a standard curve utilizing recombinant proteins as provided by the manufacturer, a plate-control (invariant sample from one discrete sample, from a single extraction and single thaw), and plate position for each subject was fixed for each individual ELISA. Quantification and normalization was performed in the following sequence: 1) quantification to standard curve measured in ng/ul; 2) scaled to plate-control sample and 3) normalized to total histone protein. Intra-assay coefficient of variation (within-plate) was calculated as an average for all individual CVs for the duplicate reading of the plate-control sample and was 7%; inter-assay (between-plates) for the same plate-control was 10%.

### mRNA extraction and real time RT-PCR quantification

For both human PBMCs and mouse samples, total RNA was isolated using TRIzol reagent (Life Technologies), and treated with DNase (Ambion/Life Technologies AM1906) after extraction. Only total RNA extracts with an OD260/OD280 ratio above 1.96, indicating relatively pure RNA, were processed for RT-PCR, the remainder undergoing re-extraction [39]. Total RNA was used to prepare cDNA via the Applied Biosystems High Capacity Archive Kit (#4368814). For detection and measurement of expression, Fermentas Maxima SYBR Green/ROX qPCR Master Mix (#K0222) was used. PCR mixtures were run on a Thermo Scientific PikoReal real-time PCR System using the following conditions: 95ᵒC for 10 minutes, followed by 40 cycles of 95ᵒC for 30 seconds, 60ᵒC for one minute and 72ᵒC for one minute. Cycle threshold (CT) value was used for relative quantification. For human PBMC samples, all values were normalized to three housekeeping genes, GAPDH, TFRC and b-actin using a geometric mean, and run in triplicate [40]. Mouse mRNA samples were normalized to b-actin. Primer sequences are listed in S1 Table, with human primers (h) used in PBMC samples and mouse (m) primers used in mouse brain extracts.

### Chromatin immunoprecipitation

The fast chromatin immunoprecipitation (ChIP) method was performed as previously published [41]. Cells were cross-linked with 16% methanol-free formaldehyde and quenched with 1M glycine. Cells were lysed in SDS lysis buffer with protease inhibitors (1% SDS, 10mM EDTA, 50mM Tris-HCl, pH 8.1) and sonicated for 20 minutes at 10%df (Covaris M220). Anti-H3K9me2 rabbit monoclonal antibody (Abcam #ab1220) was used to precipitate H3K9me2 associated DNA. The protein/DNA complexes were pulled down using agarose beads (Protein A/G PLUS-Agarose, Santa Cruz #SC-2003). To control for the non-specific antibody binding in our samples, a no-antibody immunoprecipitation was performed in tandem [42,43]. DNA was isolated using a phenol chloroform extraction. Real time PCR was used to amplify DNA promoter sequences, as listed in S1 Table. All ChIP data were normalized to both the no-antibody control and an “input” condition [44].

### Data analysis

GraphPad Prism 5.0 was used for all statistical analyses. Data are presented as mean values ± standard error of the mean. Data were statistically evaluated for significance by using a one-way ANOVA, utilizing Tukey post analysis. A probability level of *p*<0.05 was the criterion to achieve statistical significance. All data for this manuscript is stored on the online repository Dryad; DOI: https://doi.org/10.5061/dryad.15n9404.

## Results

### BIX reduces total H3K9me2 levels in cultured PBMCs

**Fig 1:**
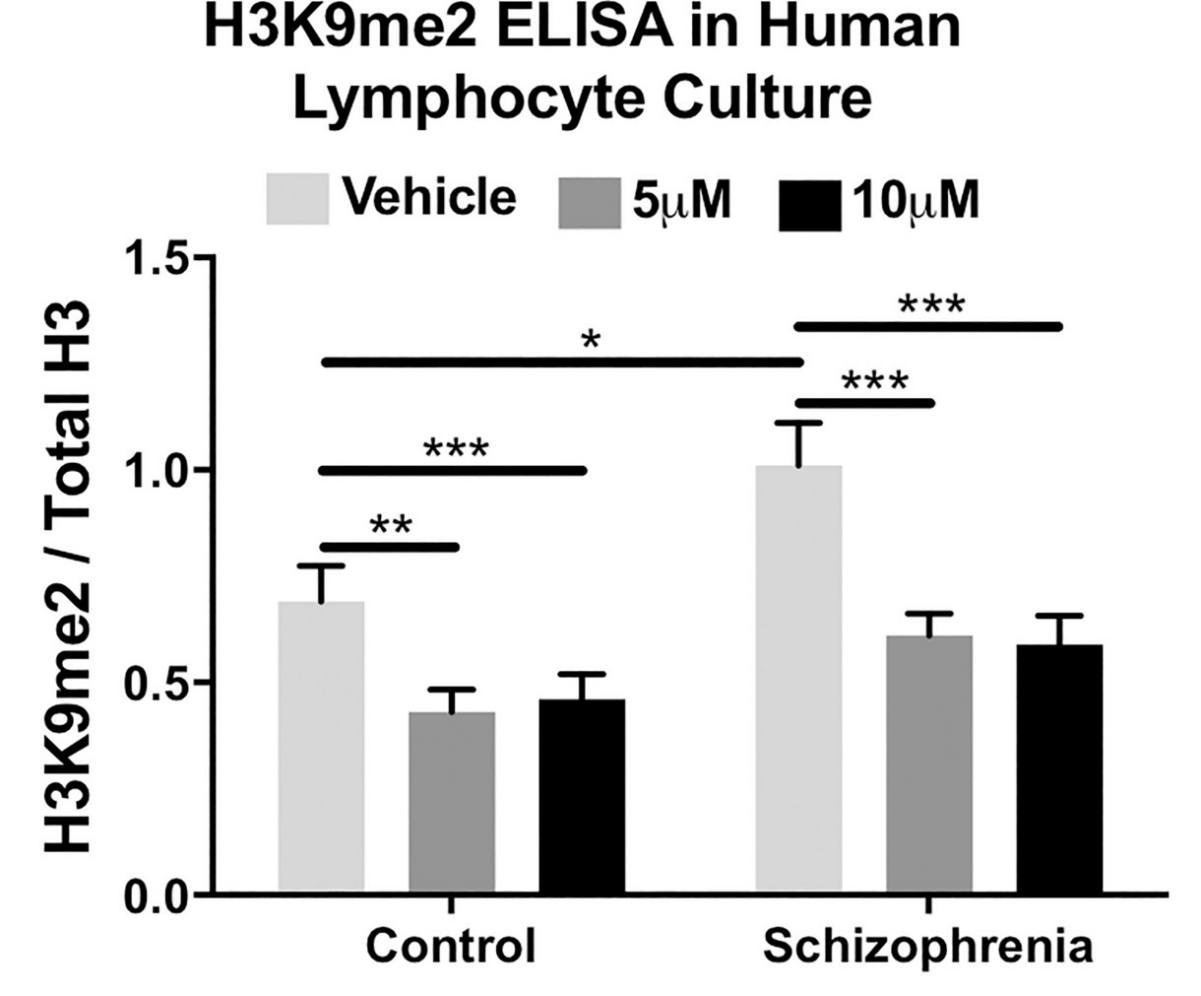
H3K9me2 levels in human PBMC culture in response to 24-hour culture with BIX-01294. BIX was able to significantly decrease H3K9me2 levels in both normal controls and participants with schizophrenia, as determined by ANOVA and Tukey post hoc analysis. *p<0.05; **p<0.01; ***p<0.001.

We sought to determine if BIX administration could alter the restrictive epigenetic modification H3K9me2 in human tissues, from both normal controls and participants with schizophrenia. Previous literature demonstrated increased H3K9me2, G9a and GLP levels in PBMCs from participants with schizophrenia. Indeed, twenty-four hour culture with BIX significantly decreased total H3K9me2 levels in peripheral mononuclear cells from both controls (ANOVA; F_2,69_ = 10.8, *p*<0.001) and participants with schizophrenia (ANOVA; F_2,69_ = 23.5, *p*<0.001). Tukey post hoc analysis revealed a significant difference between vehicle and either treatment conditions (5μM and 10μM) for both diagnostic groups (Fig 1).

### BIX alters mRNA levels in cultured PBMCs

In addition to H3K9me2 levels, we also examined the impact of BIX administration on schizophrenia candidate gene mRNA expression. BIX significantly increased mRNA expression of all four genes examined: IL-6, GAD67, NANOG and KLF4, although we found expression patterns differed by dose and diagnostic group.

**Fig 2:**
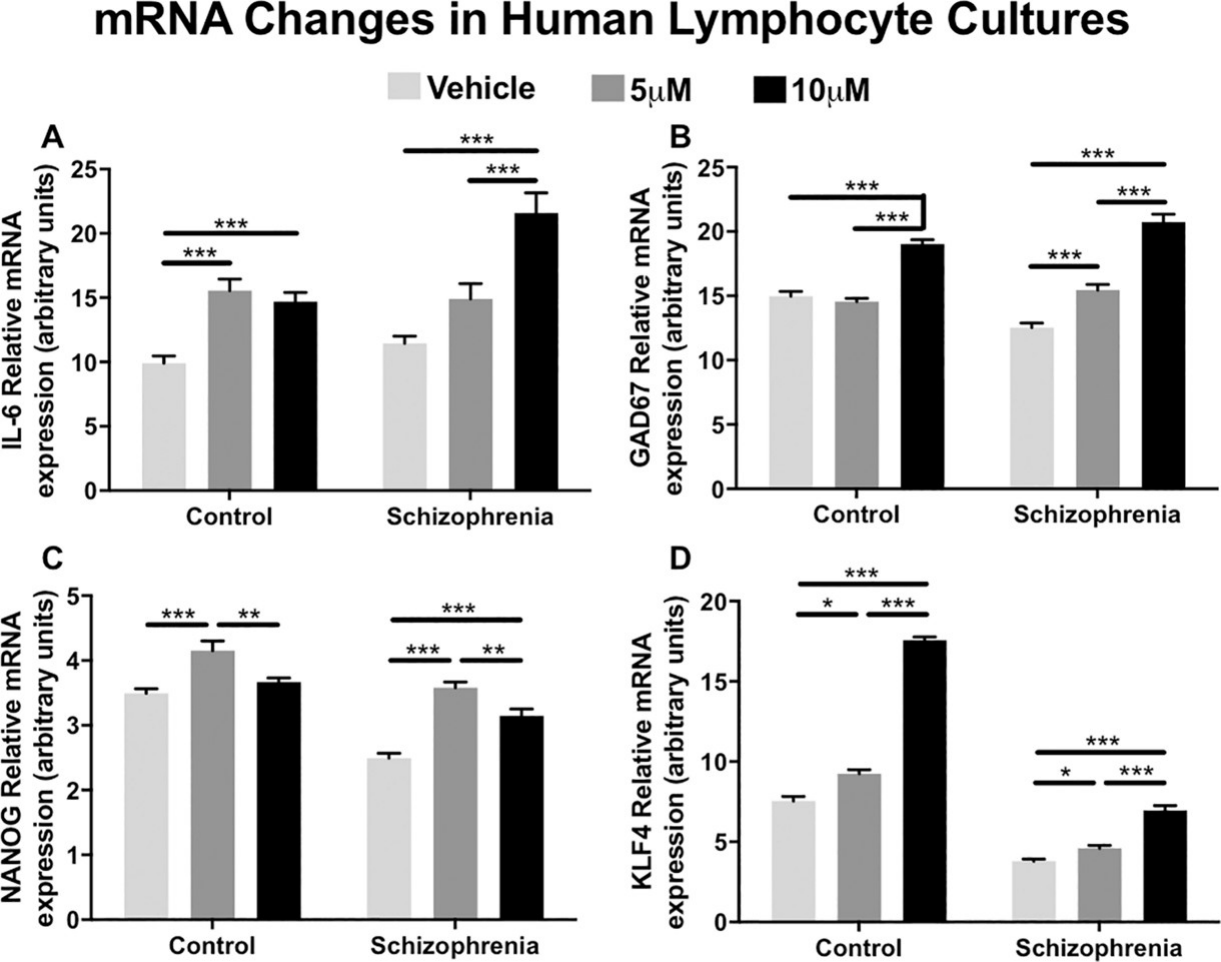
mRNA expression of selected genes in response to 24-hour culture with BIX-01294. All four genes, IL-6, GAD67, NANOG and KLF4, were all significantly upregulated in response to BIX in both normal controls and participants with schizophrenia, as determined by ANOVA and Tukey post hoc analysis. *p<0.05; **p<0.01; ***p<0.001.

BIX was able to significantly upregulate IL-6 mRNA (control: F_2,69_ = 17.2, *p*<0.001; schizophrenia: F_2,69_ = 19.4, *p*<0.001; Fig 2A), GAD67 mRNA (control: F_2,69_ = 56.2, *p*<0.001, schizophrenia: F_2,69_ = 75.1, *p*<0.001; Fig 2B), NANOG mRNA (control: F_2,69_ = 11.27, *p*<0.001, schizophrenia: F_2,69_ = 38.4, *p*<0.001; Fig 2C) and KLF4 mRNA (control: F_2,69_ = 494.6, *p*<0.001, schizophrenia, F_2,69_ = 60.9, *p*<0.001; Fig 2D) in both diagnostic groups.

### Peripheral administration of BIX reduces total H3K9me2 levels in the mouse cortex

**Fig 3:**
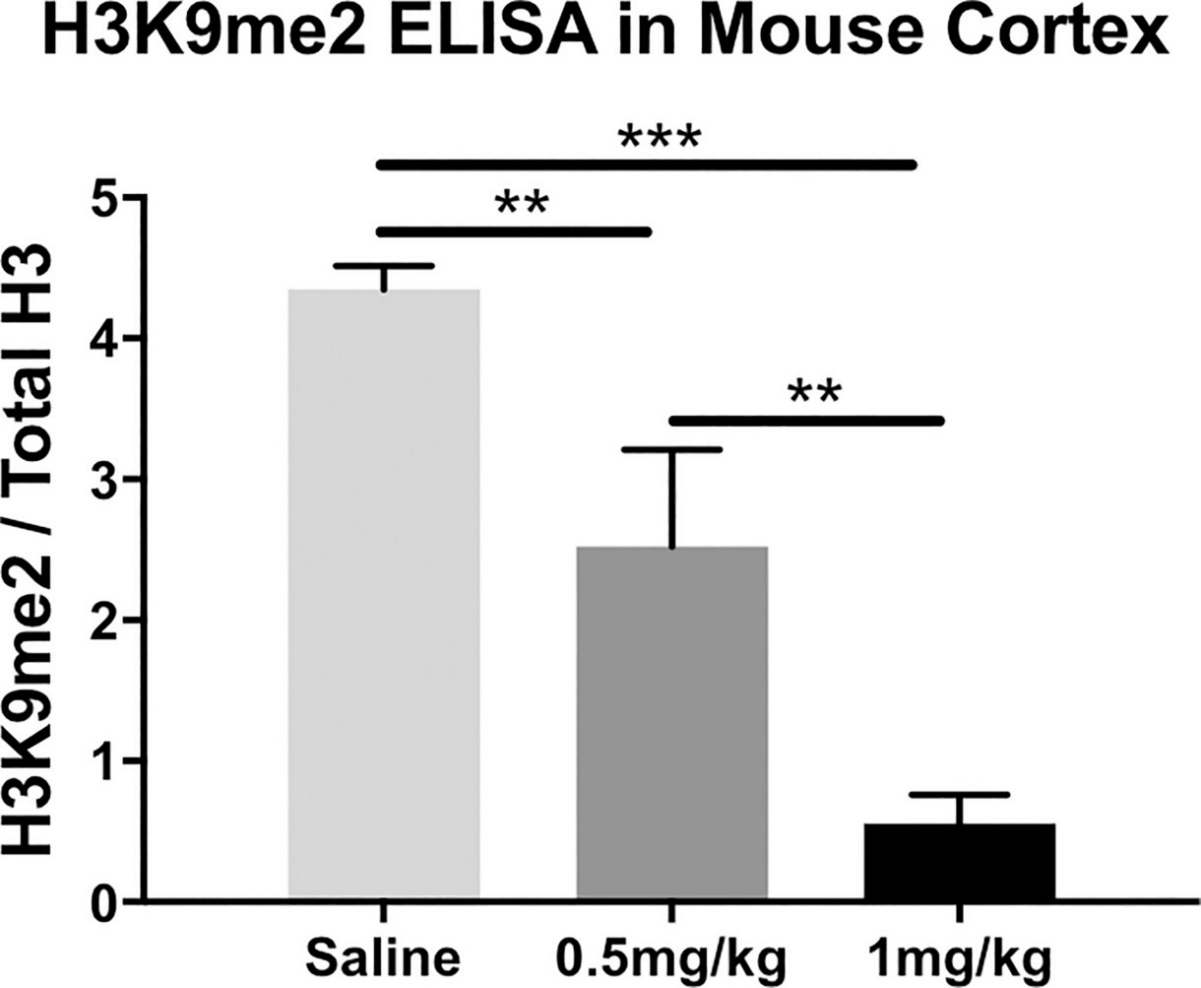
Cortical H3K9me2 levels from mice treated with weeklong intraperitoneal injections of BIX-01294. Both doses of BIX were able to significantly decrease cortical H3K9me2 levels, as determined by ANOVA and Tukey post hoc analysis. ***p<0.001.

In an initial feasibility study, we gave wild-type mice weeklong, once daily intraperitoneal (i.p.) injections of BIX (Vehicle, 0.5 or 1 mg/kg). We found that peripheral administration of BIX significantly reduced total H3K9me2 levels in cortical extracts from mice (F_2,16_ = 99.06, *p*<0.001). Tukey post hoc analysis indicated that both doses of BIX (0.5 and 1 mg/kg) significantly decreased H3K9me2 levels, with the 1 mg/kg dose being the most effective (Fig 3).

### Peripheral administration of BIX alters mRNA levels in the mouse cortex

In the feasibility study, to examine if the same group of schizophrenia candidate genes examined in the human PBMC culture had altered expression in the cortex from animals treated with BIX (Vehicle, 0.5 or 1 mg/kg), we studied cortical mRNA changes in those same genes, with the addition of two brain-specific genes: Reln and Bdnf9a. We found that BIX increased mRNA expression of five of the six genes examined.

**Fig 4:**
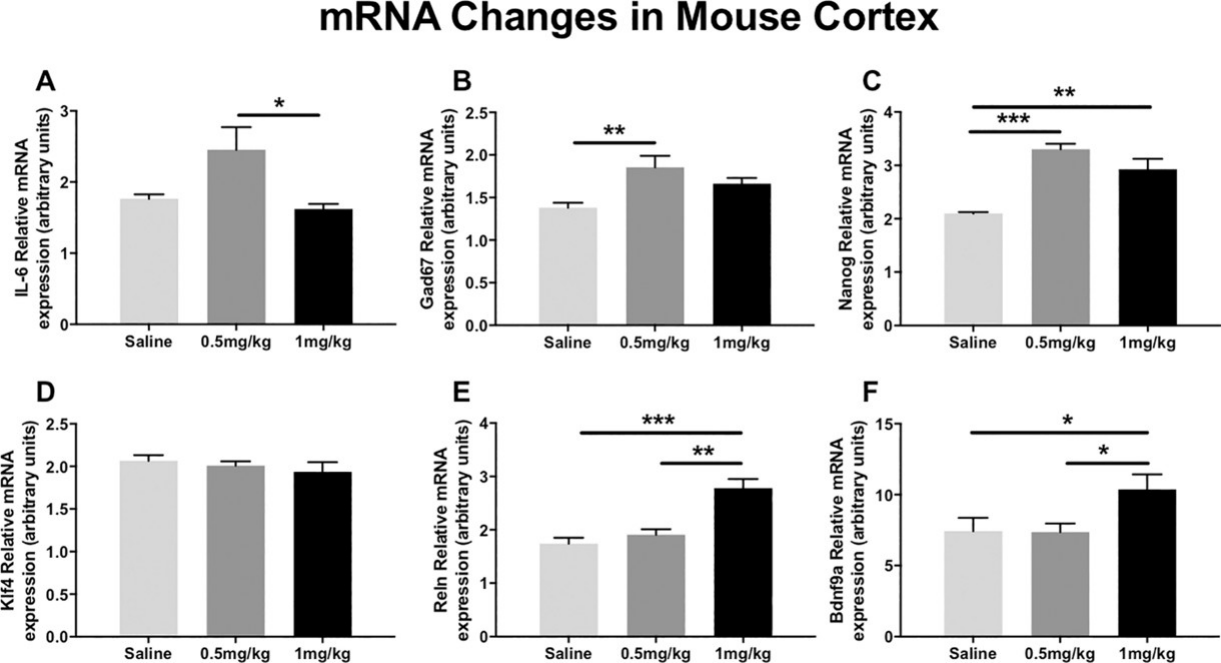
mRNA expression of known epigenetically modified genes in response to weeklong intraperitoneal injections with BIX-01294. Out of the six genes examined, the 0.5 mg/kg dose was able to significantly upregulate mRNA levels of IL-6, Gad67 and Nanog. The 1 mg/kg dose was effective at inducing mRNA levels of Nanog, Reln and Bdnf9a. Klf4 mRNA levels were unchanged. Results determined by ANOVA and Tukey post hoc analysis. *p<0.05; **p<0.01; ***p<0.001.

IL-6 (ANOVA; F_2,16_ = 5.4, *p*<0.05) mRNA was significantly elevated at the 0.5 mg/kg dose, but only when compared to the 1 mg/kg dose, not saline, as determined by post hoc analysis (Fig 4A). Gad67 (ANOVA; F_2,16_ = 6.7, *p*<0.05) mRNA was significantly upregulated in the mouse cortex after week-long peripheral BIX administration in response to 0.5 mg/kg, but not 1 mg/kg, when compared to saline, as determined by post hoc analysis (Fig 4B). Nanog mRNA was significantly upregulated (ANOVA; F_2,16_ = 23.1, *p*<0.001) in both 0.5 and 1 mg/kg conditions compared to saline, in response to BIX treatment (Fig 4C). Klf4 mRNA levels were not significantly altered in response to either week-long peripheral doses of BIX (ANOVA; F_2,16_ = 0.63, *p* = 0.5). These results run counter to the effects seen in the cultured PBMCs reported earlier (Fig 4D). Reln (ANOVA; F_2,16_ = 18.4, *p*<0.001; Fig 4E) and Bdnf9a (ANOVA; F_2,16_ = 6.1, *p*<0.01; Fig 4F) mRNA levels were also significantly increased with 1 mg/kg dose of BIX when compared to both saline and 0.5 mg/kg treatment conditions, as determined by post hoc analysis.

### Peripheral administration of BIX modifies H3K9me2 binding to select promoter regions in the mouse cortex

Based on the H3K9me2 ELISA and mRNA results presented, we sought to determine the effect of BIX on H3K9me2 promoter occupancy of the six schizophrenia candidate genes previously examined in the feasibility study. This was to characterize if mRNA changes seen were a result of decreased H3K9me2 promoter occupancy, or other epigenetic alterations. We found that BIX was able to significantly alter H3K9me2 binding to promoter regions of six selected genes: IL-6, Gad67, Nanog, Klf4, Reln and Bdnf9a.

**Fig 5:**
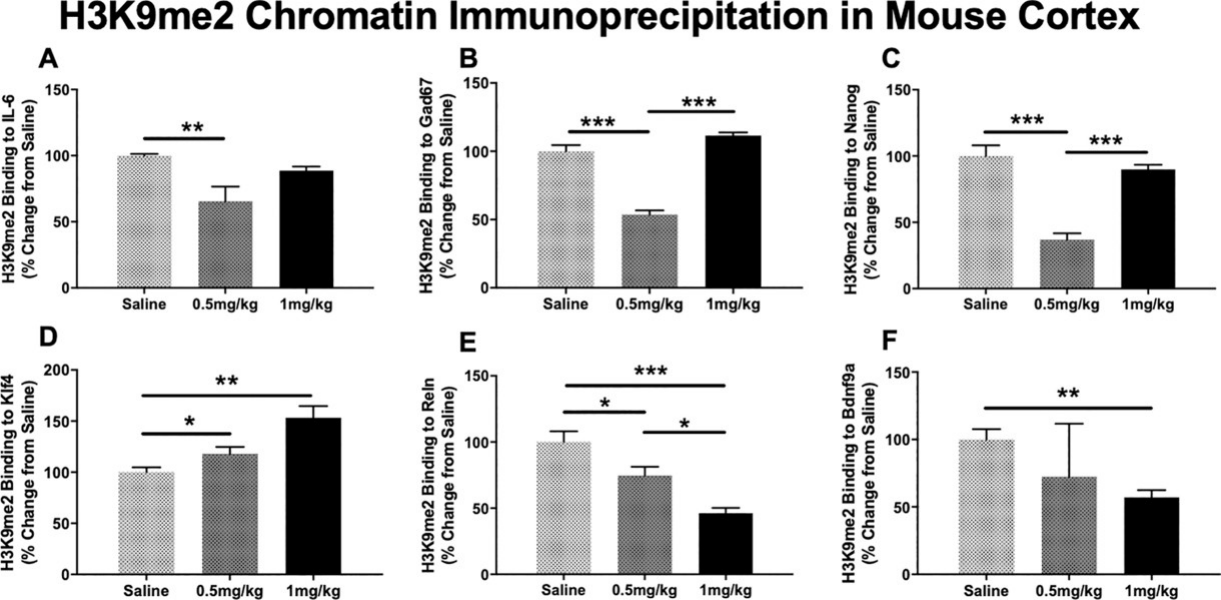
Chromatin immunoprecipitation of cortical H3K9me2 in response to intraperitoneal BIX-01294 weeklong treatment. H3K9me2 binding to the promoter regions of IL-6, Gad67, Nanog, and Reln were significantly decreased at the 0.5 mg/kg dose. The 1 mg/kg dose was also able to significantly decrease H3K9me2 binding to the promoter regions of Reln and Bdnf9. There were increases in H3K9me2 binding to the promoter region of Klf4 in response to BIX treatment, as determined by ANOVA and Tukey post hoc analysis. *p<0.05; **p<0.01; ***p<0.001.

H3K9me2 binding to the promoter regions of IL-6 (ANOVA; F_2,16_ = 6.86, *p*<0.05; Fig 5A), Gad67 (ANOVA; F_2,16_ = 80.3, *p*<0.001; Fig 5B) and Nanog (ANOVA; F_2,16_ = 34.7, *p*<0.001: Fig 5C) were significantly decreased after a week-long treatment of BIX. Tukey post hoc analysis indicated that only the 0.5 mg/kg dose resulted in a significant decrease of H3K9me2 binding for each of these genes. This pattern mirrors the significant increase in mRNA seen in cultured lymphocytes.

BIX significantly increased H3K9me2 binding to the promoter region of Klf4 (ANOVA; F_2,16_ = 11.24, *p*<0.01; Fig 5D), also reflective of the lack of mRNA changes demonstrated above.

The week-long treatment with 1 mg/kg BIX significantly decreased H3K9me2 binding to the promoter regions of both Reln (ANOVA; F_2,16_ = 17.3, *p*<0.001; Fig 5E) and Bdnf9a (ANOVA; F_2,16_ = 4.56, *p*<0.05; Fig 5F), which is consistent with the previously reported increases in mRNA levels at the 1 mg/kg dose.

These initial feasibility studies demonstrate that peripherally administered BIX was able to significantly alter the epigenetic modification H3K9me2, both globally and at specific promoters, in the cortex. BIX was also able to significantly increase the RNA transcripts we examined, chosen for their role in schizophrenia etiology. We also saw no significant indicators of distress, including weight loss (S1 Fig), or decreases in grooming.

### Peripheral administration of BIX modifies BDNF9a mRNA levels in mutant animals

BDNF is reduced in participants with schizophrenia, and increasing protein levels may have beneficial clinical outcomes [30]. In mouse cortex, we demonstrated that BIX was able to significantly upregulate Bdnf mRNA levels through decreases in promoter binding of the restrictive histone modification H3K9me2. Thus, we chose to further examine the ability of BIX to rescue decreased Bdnf gene expression in conditional knockdown animals.

**Fig 6:**
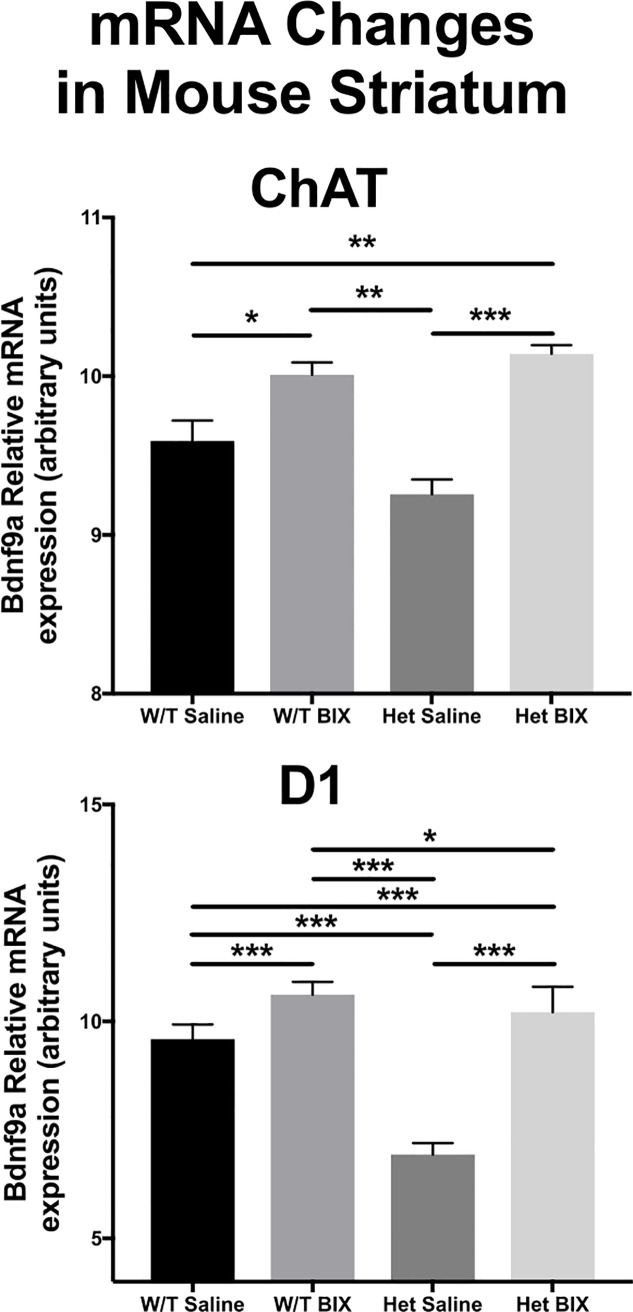
Bdnf9a mRNA levels from the striatum of three different mouse conditional knockdown lines. BIX was able to significantly rescue Bdnf mRNA expression in response to conditional knockdown using heterozygous BDNF conditional knockdown mice, bred with either ChAT or D1-*cre* animals in the striatum. *p<0.05; **p<0.01; ***p<0.001.

We took advantage of the cre-lox system to selectively knock down BDNF in two different neuronal cell types: Choline Acetyltransferase (ChAT) and dopamine receptor 1 (D1) expressing neurons. We focused on the cell types found in the striatum, as the striatum plays a role in the cognitive and negative symptoms of schizophrenia, symptoms demonstrated to be correlated with increases in the histone methyltransferases G9a and GLP mRNA levels [20]. Adult male mice were given a once daily i.p. injection of BIX (1 mg/kg), or vehicle (saline) for one week and Bdnf9a mRNA levels were examined in specific brain regions. The 1 mg/kg dose was selected for its ability to significantly upregulate Bdnf expression in the feasibility study (Fig 4). There was a rescue of Bdnf9a mRNA, to the levels of wild-type BIX treated animals in the ChAT-*cre* (ANOVA; F_3,59_ = 5.26, *p*<0.001; Fig 6A) and the D1-*cre* Bdnf conditional knockdown mice (ANOVA; F_3,39_ = 18.22, *p*<0.001; Fig 6B), similar to results seen in the feasibility study.

## Discussion

BIX-01294 (BIX) is a recently developed drug that has been demonstrated to decrease levels of the restrictive histone modification H3K9me2 through pharmacological inhibition of the histone methyltransferases G9a and GLP [45,46]. This manuscript aimed to advance our understanding of the role histone methyltransferase inhibitors could potentially play in human pharmacotherapy by examining the efficacy of this drug in both a clinical sample and in mammalian brain tissue following peripheral administration.

We demonstrate that BIX significantly reduces H3K9me2 levels and correspondingly increases mRNA levels in both *in vitro* and *in vivo* models: peripheral blood mononuclear cells (PBMC) from both normal controls and participants with schizophrenia and in mouse brain. First we examined the effects of BIX on cultured PBMCs taken from both normal controls and participants with schizophrenia. Similar to previously published results, [20] H3K9me2 levels were significantly elevated in PBMCs from participants with schizophrenia when compared to normal controls. Twenty-four hour culture with BIX significantly decreased H3K9me2 levels in both groups. This effect is strikingly different than the attenuated alterations in levels of acetylated histones in response to a histone deacetylase inhibitor in PBMCs from participants with schizophrenia [15,47]. This suggests that pharmacologically targeting histone methyltransferases may be clinically useful, as a restrictive epigenome is associated with schizophrenia symptoms that our current antipsychotics do not treat.

To identify if there were alterations in gene transcription as a result of BIX-induced differences in H3K9me2 levels, we profiled four genes, IL-6, GAD67, NANOG and KLF4. IL-6 is an immunity factor consistently demonstrated to be elevated in participants with schizophrenia [48,49]. In our cohort, participants with schizophrenia had higher IL-6 mRNA levels at baseline, with BIX treatment significantly increasing these levels. These results seem to coincide with the theory of a hyperactive immune complex seen in schizophrenia, which may not be clinically beneficial [50]. Downregulation of GAD67 is a canonical finding critical to the GABAergic neuropathology theory of schizophrenia [51]. In both the schizophrenia and control samples, BIX significantly upregulated GAD67 mRNA levels. NANOG and KLF4 are developmental transcription factors previously demonstrated to be influenced by administration of the histone deacetylase inhibitor valproic acid [52]. Additionally, in human adipose-derived mesenchymal stem cells, BIX treatment increases NANOG mRNA levels, but not KLF4 [53]. In our sample, BIX upregulated both NANOG and KLF4 mRNA levels for both the clinical and control samples, perhaps demonstrating a cell-type dependent action, as our mouse cortex samples mirrored the expression patterns seen in mesenchymal stem cells. In sum, these experiments demonstrated that in culture, BIX upregulates genes commonly observed to be downregulated in schizophrenia.

We next demonstrated that peripherally administered BIX reduced both global and promoter-specific H3K9me2 protein levels and increase mRNA levels in the mouse cortex. This suggests that peripheral BIX treatment can augment epigenomic modifications and gene expression in the mammalian brain, thus establishing it’s potential as a therapeutic agent. Six genes were chosen for both mRNA and ChIP studies; the same four genes examined in the PBMC samples (IL-6, Gad67, Nanog, and Klf4) with an additional two genes primarily expressed in cortex and not in PBMCs (Reln and Bdnf9a). Reln is another canonical schizophrenia candidate gene, with increased DNA promoter methylation, and decreased mRNA levels found in participants with schizophrenia [54].

Bdnf expression is downregulated in schizophrenia [3]. Given that BDNF is involved in synapse regulation, learning and memory, BDNF deficits seen in schizophrenia may lead to negative and cognitive symptoms [3]. Because BIX treatment significantly increased Bdnf9a mRNA levels, we sought to determine if BIX could rescue impaired neural Bdnf expression using a mouse conditional knockdown model. BIX inhibits the action of GLP and G9a, enzymes that catalyze the H3K9me2 modification. In humans, both enzymes and their corresponding H3K9me2 modification are correlated with the negative and cognitive symptoms of schizophrenia [20]. We selected the striatum, as this brain region is linked to the negative and cognitive symptoms of schizophrenia [28,55]. The striatum is composed of four primary cells types: Medium spiny neurons (MSNs) that express either the D1 or D2 families of dopamine receptors, cholinergic interneurons that express choline acetyltransferase (ChAT) interneurons, and GABAergic interneurons. We selected the D1, and ChAT expressing neurons as these cell types have each been associated with the negative and cognitive symptoms of schizophrenia, as opposed to the D2 system, which is targeted by antipsychotic medications, and thus the positive symptoms of schizophrenia.

Abnormalities in the cholinergic system are seen in schizophrenia, with the majority of findings indicating decrements in cholinergic neurotransmission [56,57]. The antipsychotic medications olanzapine and clozapine modulate cholinergic neurotransmission, and have both the greatest efficacy in treatment-resistant schizophrenia, and reductions in severity of negative symptoms [58]. Additionally, nicotine alters cholinergic signaling, increases global BDNF levels, and is highly used in persons with schizophrenia [59,60]. Nicotine use improves many cognitive abnormalities in schizophrenia, including attention and memory tasks [61]. Dopaminergic dysregulation is involved in the pathology of schizophrenia [62]. Persons with schizophrenia exhibit impairments with reward-associative, but not punishment-associative learning, an effect that is replicated with D1 receptor expression suppression in the striatum of mice [63,64]. We found that BIX rescued mRNA levels of Bdnf9a in the striatum from ChAT and D1 conditional knockdown animals, with expression levels restored to near wild-type levels from the single intact allele. These findings are interesting, and may indicate their use in mitigating the Bdnf downregulation seen in schizophrenia, which warrants further research when attempting to translate these findings back into a clinical population.

There are several limitations of the current study that should motivate future research. While this study does not contain a radiolabeling aspect to investigate if BIX crosses the blood brain barrier, we do show several lines of evidence that peripherally administered BIX alters epigenetic marks and gene expression in the central nervous system. Additionally, this is an experimental drug, with little research into the physical tolerability and side effects of administration. We did not observe distress in the animals, as their weight did not significantly differ between treatment groups. Future directions include identifying if BIX is able to modulate schizophrenia-like behaviors in animals, including prepulse inhibition, social interaction or more cognitive-based behaviors including memory, and cognitive function. Both the literature [45,46] and these preliminary studies indicate that BIX is effective at significantly decreasing H3K9me2 levels *in vitro*. Thus, further research into the effectiveness, tolerability and viability of use of this drug in mammals needs to be examined.

## Conclusions

In sum, we demonstrate the effectiveness of BIX at reducing H3K9me2 levels and increasing gene transcription in the brain when given peripherally. These results suggest the possibility of a methylated histone based co-therapy that may be a robust method for modifying the dysregulated epigenome in clinical populations [47].

## Supporting information

S1 Fig Recorded mouse weights during the feasibility study in response to a once daily intraperitoneal injection (i.p.) of BIX-01294 (vehicle, 0.5 or 1 mg/kg) for one week (n = 5 per condition). Weights between conditions were not statistically significant, as determined by ANOVA and Tukey post hoc analysis.
(TIFF)

S1 Table Real-time RT-PCR mRNA primer sequences. Human primers used in peripheral blood mononuclear samples are indicated by a leading H, and mouse primers used in mouse brain extracts are indicated by a leading M.
(DOCX)
