# Supplementary figures and images for: Examining the effects of the histone methyltransferase inhibitor BIX-01294 on histone modifications and gene expression in both a clinical population and mouse models
Source: PLoS One. 2019 Jun 11;14(6):e0216463. doi: 10.1371/journal.pone.0216463 (PMC6559633; doi:10.1371/journal.pone.0216463)

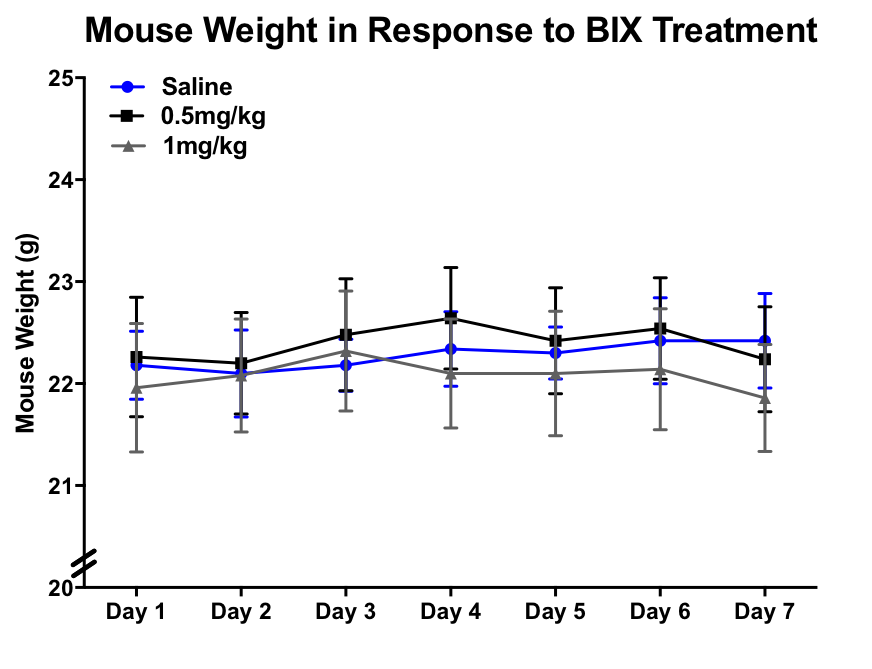

Supplement: S1 Fig — Weights between conditions were not statistically significant, as determined by ANOVA and Tukey post hoc analysis. (TIFF) [file pone.0216463.s001.tiff]
