# Supplementary material for: Examining the effects of the histone methyltransferase inhibitor BIX-01294 on histone modifications and gene expression in both a clinical population and mouse models
Source: PLoS One. 2019 Jun 11;14(6):e0216463. doi: 10.1371/journal.pone.0216463 (PMC6559633; doi:10.1371/journal.pone.0216463)
Supplement: S1 Table — Human primers used in peripheral blood mononuclear samples are indicated by a leading H, and mouse primers used in mouse brain extracts are indicated by a leading M. (DOCX) [file pone.0216463.s002.docx]

**S1 Table - Primer Sequences**

| **Primer Name** | **Left Primer** | **Right Primer** |
| --- | --- | --- |
| **mRNA Expression Primers** | | |
| H_IL-6 | AAAGAGGCACTGGCAGAAAA | AGCTCTGGCTTGTTCCTCAC |
| H_GAD67 | GGAACTAGCGAGAACGAGGA | ACGAAGATGGGGTCGAAGAC |
| H_NANOG | AACTGGCCGAAGAATAGCAA | ACTGGATGTTCTGGGTCTGG |
| H_KLF4 | CCCCGTGTGTTTACGGTAGT | GTTCCCATCTCAAGGCACAC |
| H_GAPDH | CGAGATCCCTCCAAAATCAA | TTCACACCCATGACGAACAT |
| H_TFRC | CACCAACCGATCCAAAGTCT | AAAATCCGGTGTAGGCACAG |
| H_βActin | TGAAGGTAGTTTCGTGGATGC | TCCCTGGAGAAGAGCTACGA |
| M_IL-6 | AACGATGATGCACTTGCAGA | CTCTGAAGGACTCTGGCTTTG |
| M_Gad67 | TCCAAGAACCTGCTTTCCTG | GGTGGAGCGATCAAATGTCT |
| M_Nanog | CCAAAGGATGAAGTGCAAGC | CCAGATGCGTTCACCAGATA |
| M_Klf4 | CCTGTGTGTTTGCGGTAGTG | CCAAAGAGGGGAAGAAGGTC |
| M_Reln | GCCTCCAATGCTCTGAGAAG | GTGCTCATTTCCCTGCACAT |
| M_Bdnf9a | GCAGCTGGAGTGGATCAGTAA | TGGTCATCACTCTTCTCACCTG |
| M_βActin | ACATCTGCTGGAAGGTGGAC | TTGCTGACAGGATGCAGAAG |
| **Chromatin Immunoprecipitation Primers** | | |
| M_IL-6 | AAGTCAGCCCAAGGGATCTT | TCCCAGGCATTTTAGGAGTG |
| M_Gad67 | TCCGGATCTCTCCCTTCTTC | TCGGCTCTGTCACAGGAGTA |
| M_Nanog | AGCTCTTACAATTCCTCTCCCGGACGGTT | AGCCCAGCAAACTACCTTCACTAGGCCAAA |
| M_Klf4 | GAATTGGGACACTGGGACAC | AGCGGTTCCCCACTAGTCTT |
| M_Reln | GGAAAAGGATGGAAAGATGC | TCTCGCCTTTCTTTTTCCTG |
| M_Bdnf9a | CATGAGACCGGGCAAGTC | CCTTGGGAGGAATGTGTGAT |
